# Accuracy of Consumer Wearable Heart Rate Measurement During an Ecologically Valid 24-Hour Period: Intraindividual Validation Study

**DOI:** 10.2196/10828

**Published:** 2019-03-11

**Authors:** Benjamin W Nelson, Nicholas B Allen

**Affiliations:** 1 Department of Psychology University of Oregon Eugene, OR United States; 2 Center for Digital Mental Health University of Oregon Eugene, OR United States

**Keywords:** electrocardiography, Apple Watch 3, digital health, Fitbit Charge 2, heart rate, mobile health, passive sensing, photoplethysmography, wearables

## Abstract

**Background:**

Wrist-worn smart watches and fitness monitors (ie, wearables) have become widely adopted by consumers and are gaining increased attention from researchers for their potential contribution to naturalistic digital measurement of health in a scalable, mobile, and unobtrusive way. Various studies have examined the accuracy of these devices in controlled laboratory settings (eg, treadmill and stationary bike); however, no studies have investigated the heart rate accuracy of wearables during a continuous and ecologically valid 24-hour period of actual consumer device use conditions.

**Objective:**

The aim of this study was to determine the heart rate accuracy of 2 popular wearable devices, the Apple Watch 3 and Fitbit Charge 2, as compared with the gold standard reference method, an ambulatory electrocardiogram (ECG), during consumer device use conditions in an individual. Data were collected across 5 daily conditions, including sitting, walking, running, activities of daily living (ADL; eg, chores, brushing teeth), and sleeping.

**Methods:**

One participant, (first author; 29-year-old Caucasian male) completed a 24-hour ecologically valid protocol by wearing 2 popular wrist wearable devices (Apple Watch 3 and Fitbit Charge 2). In addition, an ambulatory ECG (Vrije Universiteit Ambulatory Monitoring System) was used as the gold standard reference method, which resulted in the collection of 102,740 individual heartbeats. A single-subject design was used to keep all variables constant except for wearable devices while providing a rapid response design to provide initial assessment of wearable accuracy for allowing the research cycle to keep pace with technological advancements. Accuracy of these devices compared with the gold standard ECG was assessed using mean error, mean absolute error, and mean absolute percent error. These data were supplemented with Bland-Altman analyses and concordance class correlation to assess agreement between devices.

**Results:**

The Apple Watch 3 and Fitbit Charge 2 were generally highly accurate across the 24-hour condition. Specifically, the Apple Watch 3 had a mean difference of −1.80 beats per minute (bpm), a mean absolute error percent of 5.86%, and a mean agreement of 95% when compared with the ECG across 24 hours. The Fitbit Charge 2 had a mean difference of −3.47 bpm, a mean absolute error of 5.96%, and a mean agreement of 91% when compared with the ECG across 24 hours. These findings varied by condition.

**Conclusions:**

The Apple Watch 3 and the Fitbit Charge 2 provided acceptable heart rate accuracy (<±10%) across the 24 hour and during each activity, except for the Apple Watch 3 during the daily activities condition. Overall, these findings provide preliminary support that these devices appear to be useful for implementing ambulatory measurement of cardiac activity in research studies, especially those where the specific advantages of these methods (eg, scalability, low participant burden) are particularly suited to the population or research question.

## Introduction

### Background

Wrist-worn smartwatches and fitness monitors or wearables have been widely adopted by consumers and are currently gaining increased attention by researchers for their potential contribution to digital measurement of health, especially in *big data* studies as these devices are scalable, unobtrusive, and potentially provide greater ecological validity (ie, the degree to which a research design matches naturalistic environments to generalize results to real-life settings), as compared with laboratory studies. These devices contain a multitude of sensors, often including an optical sensor that uses photoplethysmography (PPG) that allows these devices to collect pulse rate or volumetric changes in blood profusion that act as a surrogate for heart rate (HR). Although often used interchangeably, it is important to note that pulse rate and HR are 2 different physiological signals [1], with pulse rate representing the rate of change in blood pressure because of the ventricular ejection of blood, whereas HR represents the rate of heart contraction as indexed by heart electrical impulses. As such, the goal of wearable HR accuracy validation studies is to assess that device measurements, such as those between wearables and a reference method (ie, electrocardiogram; ECG), are not outside of clinically important limits of agreement (LoA), so that devices can supplement, replace, or even be used interchangeably [2].

Recently, there have been a variety of studies that have examined the accuracy of wearable PPG sensors as compared with ECG [3-9], polar chest straps [10,11], or pulse oximeters [12] across various controlled laboratory conditions, including sitting, treadmill protocols for walking and running, cycling, weight training, and sleeping. The current gold standard reference method for assessing HR is the ECG, which highlights the limitations of many studies that have utilized chest straps [10,11] or pulse oximeters [12], which themselves contain a degree of error when compared with ECG. Therefore, many studies are comparing wearable HR accuracy with suboptimal comparison methods, which likely undermine findings. Below, we primarily reviewed the existing wearable HR literature that has used an ECG as the comparison method.

Previous research comparing wearables with the gold standard ECG, which uses electrodes to measure cardiac muscular contractions from electrical activity of the heart, has shown that that wearables underestimate absolute HR as compared with reference methods [3-4,6,8-11,13]. Prior research has also shown that the Apple Watch has greater accuracy than Fitbit devices [7-9]. Specifically, prior research has found that the Apple Watch has lower overall error [3,7,10], lowest mean difference [8], and higher agreement with ECG than Fitbit devices [3,9], but that wearables’ accuracy depends on activity [5]. Research has shown that at rest, wearables can perform similarly to an ECG but not with moderate exercise [14]. There has been a substantial amount of research that has shown that wearable devices are more accurate during rest and low intensity exercise as compared with exercises at higher intensity [3,5,9,15-17], which may be because of the position of the device during rest [18] and less movement of the wearable device around the wrist at rest, although this is not found in all studies [7,10-11,19]. Specifically, 1 study found that there was not a significant difference in HR accuracy across baseline or vigorous activity [10], whereas a second study found that HR accuracy was highest during running––a very intense activity [19], and a third found that walking, running, and cycling were more accurate for some devices than sitting [7]. Therefore, it is possible that activity intensity may be less important to device accuracy than the degree of erratic wrist movements performed during physical activity, which tend to co-occur with more vigorous physical activity.

### Four Challenges Limiting Progress for Wearable Heart Rate Accuracy

Currently, prior research has greatly improved our understanding of wearable HR accuracy, but there remain 4 challenges that limit progress in this area. First, as mentioned above, many studies lack an appropriate comparison method by opting to utilize chest straps [10,11] or pulse oximeters [12], rather than an ECG, which themselves contain a degree of error when compared with ECG. Therefore, many studies are comparing wearable HR accuracy with suboptimal comparison methods, which likely undermine findings. Second, wearable manufacturers use proprietary algorithms to translate PPG signals to HR measurements. These algorithms are likely altered with firmware updates, yet most studies fail to report firmware information. This may lead to poor reproducibility as 2 studies investigating the same device with different firmware versions might actually come to different conclusions even if all other variables are held constant. Third, almost all prior studies have utilized laboratory paradigms, rather than naturalistic settings. Recent research has called for the test of devices in the *setting appropriate for intended use* [20]. Although controlled laboratory settings are important for maintaining experimental control, this design involves a trade-off that often creates an artificial environment during which individual behaviors may deviate from that in naturalistic settings of lived daily experience. For example, laboratory settings tend to test specific movements within predetermined time frames, whereas consumers use wearables in naturalistic settings that often involve more variable and sporadic movements, which may not be accurately captured during laboratory paradigms. As such, the accuracy of wearables in controlled settings may deviate from accuracy during the daily living conditions of consumers. The 2 studies that were identified to have been conducted in more naturalistic settings have either occurred within a medical setting [6], which inherently does not capture the vast majority of consumer device use conditions, or only collected a maximum of 6 hours of free-living nonsleep conditions without the use of a gold standard ECG as a reference method [21]. Finally, the speed of wearable technological advancements often outpaces the typical research cycle [22], making it very difficult for studies to validate each new iteration of wearables. This calls for novel rapid response designs to quickly assess initial wearable HR accuracy in order for the research cycle to keep pace with technological advancements.

This study addresses each of the current limitations in wearable studies as it (1) uses a gold standard comparison method for movement within daily life––ambulatory ECG, (2) reports firmware versions, (3) increases the ecological validity of wearable HR accuracy by taking place during actual consumer device use conditions across a 24-hours period, and (4) takes place within an individual, rather than a traditional group of research participants, which creates an agile and novel rapid response design to quickly assess initial wearable HR accuracy in order for the research cycle to keep pace with technological advancements. This design also controls most between-subject variability and potential confound variables, allowing wearable devices to be the only study variable that varies; thus, providing a powerful (albeit potentially less generalizable) test of device accuracy.

### Study

This study was preregistered (hypotheses and methods) with open code and data on Open Science Framework (OSF) [23]. The objective of this study was to determine the HR accuracy of 2 of the most popular wearables, the Apple Watch 3 and Fitbit Charge 2, as compared with the gold standard method for continuous recording in real-world settings––an ambulatory ECG. As mentioned above, a single-subject design was used for this initial study on the ecological validity of wearables to provide a proof-of-concept research design that will allow research cycles to keep pace with the technological advancements of wearables, while also eliminating between-subject variability. Although a single-subject design is a limitation, a recent study has highlighted the possibility that group-level findings do not apply to the individual [24] and that N of 1 trials are a promising approach to empirical decision making [25]. Furthermore, single-subject designs are being increasingly used [26-28], even in leading journals [29-31]. One strength of this design is that all potential confound variables can be held constant, except for the wearable devices; thus, providing a powerful test of accuracy of the devices *per se*.

This study hypothesized that (1) the Apple Watch 3 would be more accurate at measuring HR than the Fitbit Charge 2 when compared with an ambulatory ECG across all conditions, (2) both wearables would underestimate HR across all conditions, and (3) device measurement of HR would become increasingly inaccurate as activity intensity increased.

## Methods

### Recruitment

We investigated the accuracy of wearable HR from 2 popular devices in a single healthy human (first author) who completed a 24-hour protocol. The participant (29-years-old Caucasian male; body mass index=21.1; Fitzpatrick skin tone measure=2; right wrist (cm)=7.0; left wrist (cm)=6.5; right hand dominant) conceptualized and initiated this study, with the purpose of having the data published. Therefore, approval from the University of Oregon ethics committee was unnecessary and not obtained. The first author gave consent for collecting and using the data for study purposes.

### Study Protocol

Participant’s psychophysiology recordings began at 18:28 on day 1 and briefly stopped at 17:10 on day 2 before the run condition. Recording resumed at 17:37 for the run condition and stopped at 18:50 on day 2. Age, gender, height, and weight were used to set up both wearable devices.

### Conditions

A total of 5 daily conditions were recorded throughout the 24-hour study using a digital notebook (Google Sheets) to record activity times, resulting in 84 start and stop marker times. These included sitting, which included any seated activity; walking; running (this occurred on a treadmill to allow for a stable ambulatory ECG signal); activities of daily living (ADL), which included activities such as cleaning, brushing teeth, and cooking; and sleeping. Although prior research has excluded HR data during activity transitions, these were not excluded in this study to preserve ecological validity of device usage in real-world conditions. Therefore, although transition periods generally yield higher device error, we wanted to capture this variability as part of device accuracy in this study.

### Gold Standard Reference Method

ECG data were acquired using a standard 3-lead ambulatory ECG (Vrije Universiteit Ambulatory Monitoring System) [32,33]. ECG sampling frequencies were 1000 Hz, and HR was exported in 1-min epochs, from 00 seconds to 59 seconds.

### Wearable Devices

#### Apple Watch 3

The Apple Watch Series 3 (2017 version, Apple Inc, California, USA, v. 4.2.3) 42 mm was worn on the right wrist. According to Apple, the Apple Watch 3 samples HR approximately every 10 min or continuously during workouts using PPG with either a green light emitting diode or infrared light and photodiode sensors. In other words, during this study, the Apple Watch 3 collected HR data as would occur in real-world conditions, continuously for walking and running and approximately every 10 min during all other activities. The Apple Watch 3 was synced with the Apple Health app on the iPhone and then exported in XML format for analysis. The Apple Health Analysis GitHub repository [34] was used to convert the XML file to a data frame in R Studio to access per min data for analysis. When more than 1 heart rate measurement was collected each min during continuous HR recording for walking and running activities, the average of these measurements was used in line with prior wearable research [7].

#### Fitbit Charge 2

The Fitbit Charge 2 (2017 version, Fitbit Inc, California, USA, v. 22.55.2) was worn on the left wrist. According to Fitbit, the PurePulse PPG technology utilizes green LED light to continuously index HR. The Fitbit GitHub repository [35] was used to interact with the Fitbit app programming interface to access per minute data for analysis.

#### Error

To assess error, we used mean error (ME), mean absolute error (MAE), and mean absolute percent error (MAPE). In line with prior wearable research [3,11,23,36,37] as well as recommendations from the Association for the Advancement of Medical Instrumentation, the Consumer Technology Association [38] and the American National Standards Institute [39], we defined an acceptable error rate for a physical monitoring device to be ±10%, as this is considered an accurate threshold for medical ECG monitors. We recognize that this is more lenient than some prior health sciences research on wearable HR accuracy [7] and pedometer step counting accuracy [40,41] that have defined an acceptable error rate to be ±5%. In line with recent recommendations [37,38], we used MAPE to determine acceptable error rate. Outliers were not removed as this would interfere with determining device accuracy during consumer use conditions.

### Statistical Analysis

All analyses were performed in R (version 3.4.3) using R Studio (version 1.1.383). Scripts can be found on GitHub [34,35] and OSF [23]. Data can be found on OSF. Analyses were performed using the beats per minute (bpm) separately for each wearable device as compared with the gold standard ECG data for HR calculated as bpm.

#### Mean Error

The ME was calculated as the difference between the device measurement and the gold standard measurement.

#### Mean Absolute Error

The MAE was calculated as the average absolute distance between the device measurement and the gold standard measurement.

#### Mean Absolute Percent Error

The MAPE relative to the ECG was calculated for each wearable device by averaging the individual absolute percent errors.

#### Bland-Altman Analysis

Bland-Altman analysis and 95% LoA were calculated using the blandr [42] and BlandAltmanLeh R packages [43]. This is the recommended method to determine agreement between medical instruments [2,44], rather than other methods of agreement, because it is unlikely that devices will have an exact agreement, and therefore, the importance lies in how close pairs of observations are, as small differences between devices are unlikely to impact patient decisions [45].

#### Concordance Class Correlation

Finally, although not one of the analyses that was preregistered, we also ran concordance class correlation (CCC) analyses between the ECG and each wearable device separately across all conditions using the DescTools R Package [46] to assist in Bland-Altman plot interpretation. In line with prior wearable research [8], the strength of agreement was interpreted based on the following, weak (CCC<.5), moderate (CCC=.5-.7), and strong (CCC>.7).

## Results

### Descriptives

**Figure 1:**
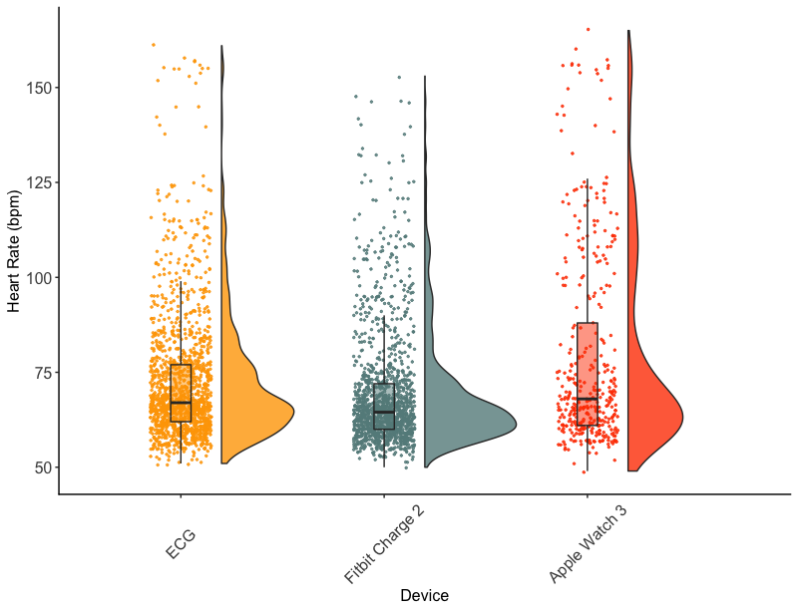
Rainbow plot of heart rate observations for electrocardiogram (ECG), Fitbit Charge 2, and Apple Watch 3. bpm: beats per minute.

**Figure 2:**
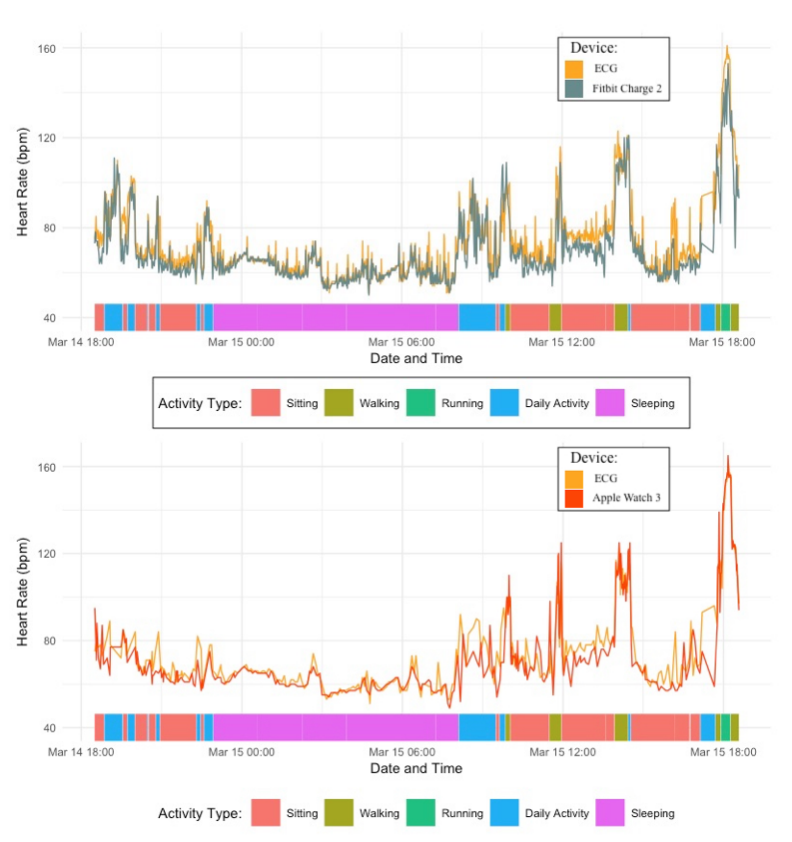
Fitbit Charge 2 (top) and Apple Watch 3 (bottom) compared to the electrocardiogram (ECG) across 24-hours. bpm: beats per minute.

The ECG collected 1424 HR observations, the Apple Watch 3 collected 394 HR observations (only collects measurements every 10 min, except during walking and running), and the Fitbit Charge 2 collected 1425 observations, resulting in a total of 3243 HR observations across devices (see Figure 1). See Table 1 for number of observations and HR descriptive statistics for each condition. See Figure 2 for descriptives of HR trajectories across the 24 hours with activity type (note that the bottom figure has less resolution as the Apple Watch 3 collected HR every 10 min, except for walking and running conditions).

**Table 1:** Heart rate descriptive statistics by condition.

Activity and device		Observations, n	Heart rate, mean (SD)	Heart rate range
**24 hours**				
	ECG^a^	1424	72.65 (16.92)	51-161
	Apple Watch 3	394	78.78 (25.74)	49-165
	Fitbit Charge 2	1446	69.10 (15.10)	50-153
**Sitting**				
	ECG	535	70.41 (7.24)	55-97
	Apple Watch 3	144	67.91 (7.69)	54-98
	Fitbit Charge 2	535	65.72 (5.51)	55-91
**Walking**				
	ECG	100	102.32 (16.87)	61-127
	Apple Watch 3	79	106.06 (15.03)	55-139
	Fitbit Charge 2	100	95.47 (17.88)	54-132
**Running**				
	ECG	22	147.82 (13.13)	104-161
	Apple Watch 3	22	149.59 (10.24)	120-165
	Fitbit Charge 2	22	133.09 (12.72)	95-153
**Activities of daily living**				
	ECG	216	84.16 (11.28)	58-115
	Apple Watch 3	34	74.94 (14.53)	52-125
	Fitbit Charge 2	214	80.38 (13.08)	56-121
**Sleeping**				
	ECG	551	61.93 (4.94)	51-78
	Apple Watch 3	110	60.60 (4.06)	49-73
	Fitbit Charge 2	551	60.82 (4.40)	50-74

^a^ECG: electrocardiogram.

### Percent Error

**Figure 3:**
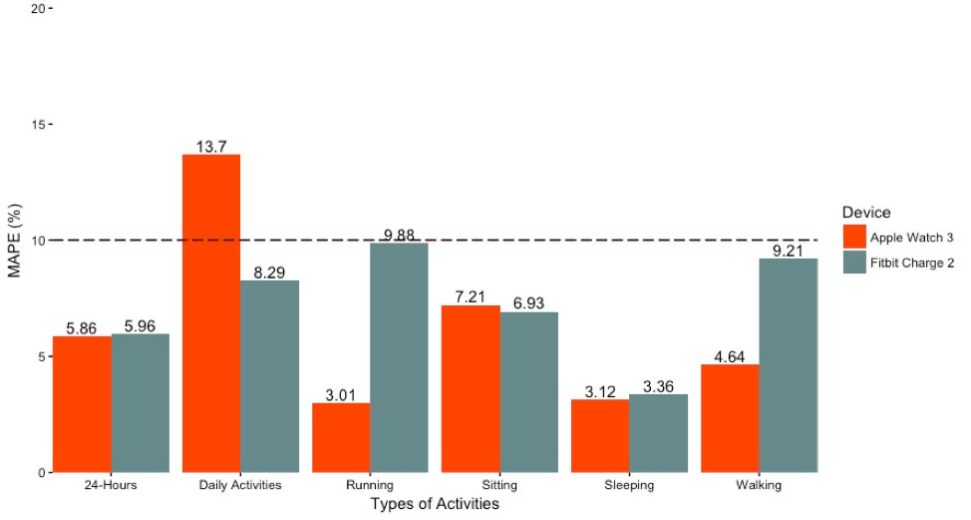
Mean absolute percent error (MAPE) by device across types of activities. Note: Horizontal line represents threshold for validity.

Overall, across the 24-hour recording, the Apple Watch 3 had a MAPE of 5.86%, whereas the Fitbit Charge 2 had MAPE of 5.96%. During sitting conditions, the Apple Watch 3 had a MAPE of 7.21%, whereas the Fitbit Charge 2 had a MAPE of 6.93%. During walking conditions, the Apple Watch 3 had a MAPE of 4.64%, whereas the Fitbit Charge 2 had a MAPE of 9.21%. During the running condition, the Apple Watch 3 had a MAPE of 3.01%, whereas the Fitbit Charge 2 had a MAPE of 9.88%. During ADL, the Apple Watch 3 had a MAPE of 13.70%, whereas the Fitbit Charge 2 had a MAPE of 8.29%. Finally,, during the sleep condition, the Apple Watch 3 had a MAPE of 3.12%, whereas the Fitbit Charge 2 had a MAPE of 3.36% (see Table 2 for percent error statistics and Figure 3 for MAPE by device across activities).

**Table 2:** Device error statistics and Bland-Altman analyses.

Activity and device		Device error			Bland-Altman analysis	
		Mean absolute error	Mean absolute percent error^a^ (%)	Mean error (SD)	Lower LoA^b^	Upper LoA
**24 hours**						
	Apple Watch 3	4.72	5.86	−1.80 (7.40)	−16.31	12.71
	Fitbit Charge 2	4.71	5.96	−3.47 (6.17)	−15.55	8.62
**Sitting**						
	Apple Watch 3	5.24	7.21	−2.47 (7.39)	−16.94	12.01
	Fitbit Charge 2	5.93	6.93	−4.69 (4.90)	−14.29	4.91
**Walking**						
	Apple Watch 3	4.77	4.64	0.11 (7.29)	−14.18	14.41
	Fitbit Charge 2	9.55	9.21	−6.85 (11.05)	−28.51	14.81
**Running**						
	Apple Watch 3	4.05	3.01	1.77 (5.90)	−9.78	13.33
	Fitbit Charge 2	14.73	9.88	−14.73 (7.67)	−29.77	0.31
**Activities of daily living**						
	Apple Watch 3	11.74	13.70	−8.50 (12.90)	−33.78	16.78
	Fitbit Charge 2	7.05	8.29	−3.73 (8.24)	−19.88	12.41
**Sleeping**						
	Apple Watch 3	1.96	3.12	−0.95 (2.78)	−6.39	4.50
	Fitbit Charge 2	2.15	3.36	−1.11 (3.20)	−7.28	5.17

^a^Validity was established as devices having a MAPE value <10%.

^a^LoA: limits of agreement.

### Bland-Altman Analysis and 95 Percent Limits of Agreement

**Figure 4:**
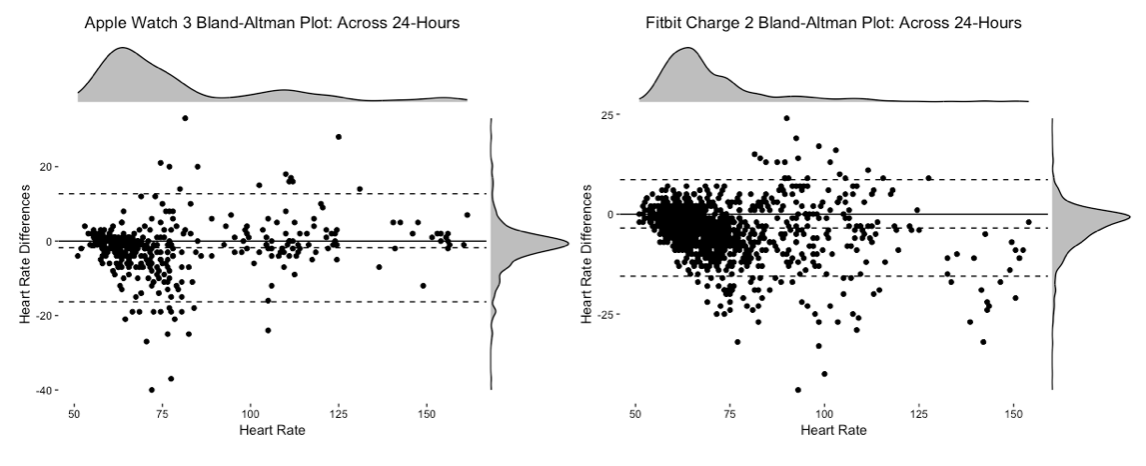
Bland-Altman plot and density plots across 24-hours of the Apple Watch 3 (left) with 394 heart rate observations and Fitbit Charge 2 (right) with 1425 heart rate observations.

Overall, across the 24-hour recording (see Figure 4), the Apple Watch 3 had an ME of −1.80 bpm (lower LoA-upper LoA: −16.31 to 12.71 bpm) and an MAE of 4.72, whereas the Fitbit Charge 2 had an ME of −3.47 bpm (lower LoA-upper LoA: −15.54 to 8.62 bpm) and an MAE of 4.71. Visual inspection of the Bland-Altman plots revealed a tendency for the Apple Watch 3 to both over- and underestimate HR values when observations were between 70 bpm to120 bpm, whereas the Fitbit Charge 2 had a tendency to underestimate HR values, particularly once HR values exceeded approximately 80 bpm (see Table 3 for Bland-Altman statistics).

**Figure 5:**
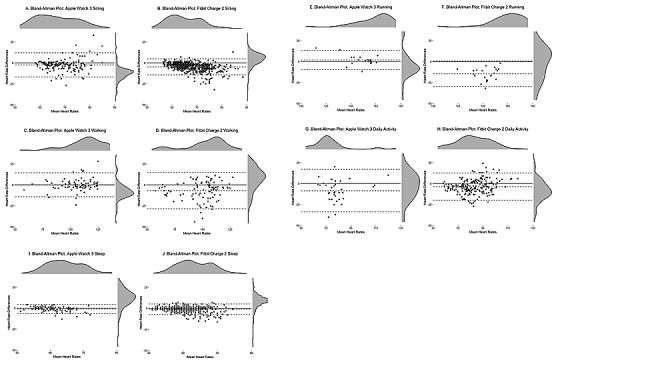
Bland-Altman plots by daily activity. Left: Apple Watch 3 during sitting; right: Fitbit Charge 2 during sitting.

**Figure 6:**
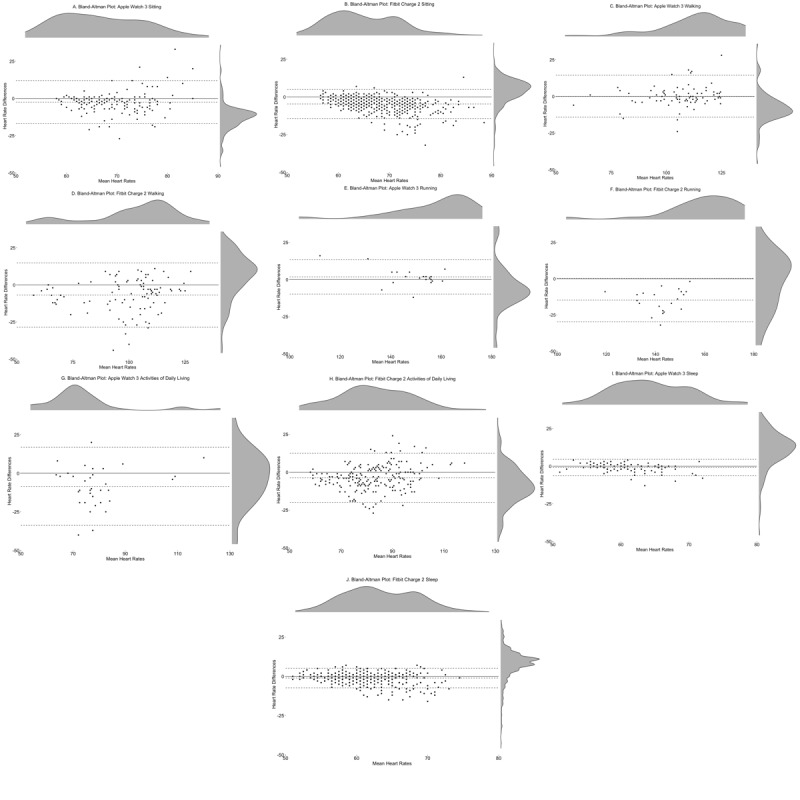
Bland-Altman plots by daily activity. Left: Apple Watch 3 during walking; right: Fitbit Charge 2 during walking.

**Figure 7:**
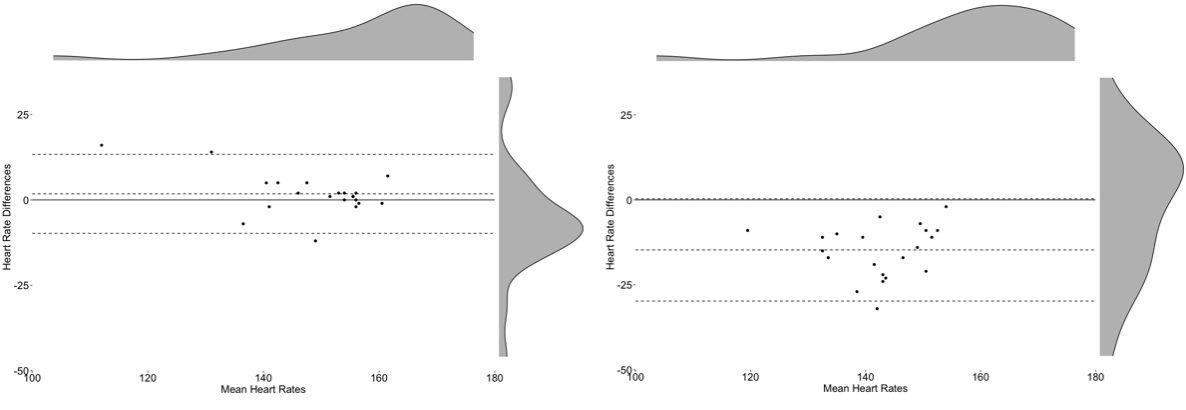
Bland-Altman plots by daily activity. Left: Apple Watch 3 during running; right: Fitbit Charge 2 during running.

**Figure 8:**
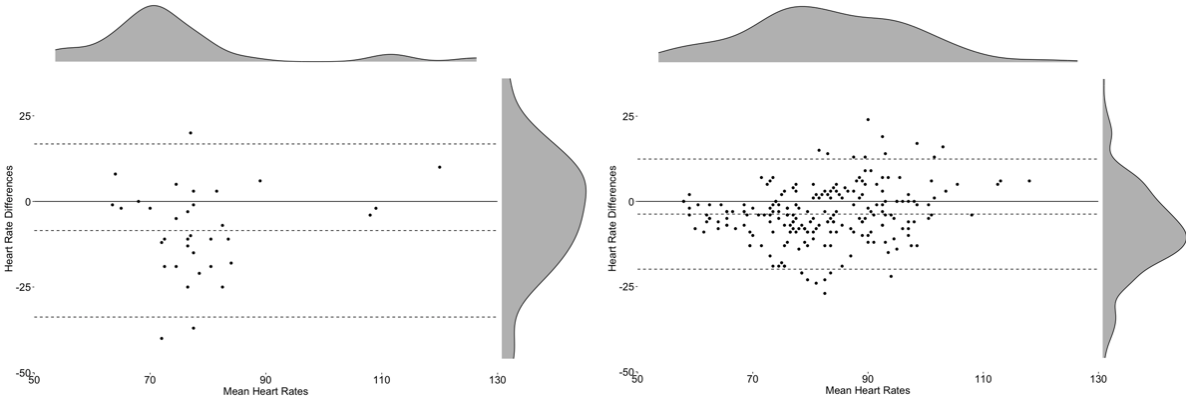
Bland-Altman plots by daily activity. Left: Apple Watch 3 during activities of daily living; right: Fitbit Charge 2 during activities of daily living.

**Figure 9:**
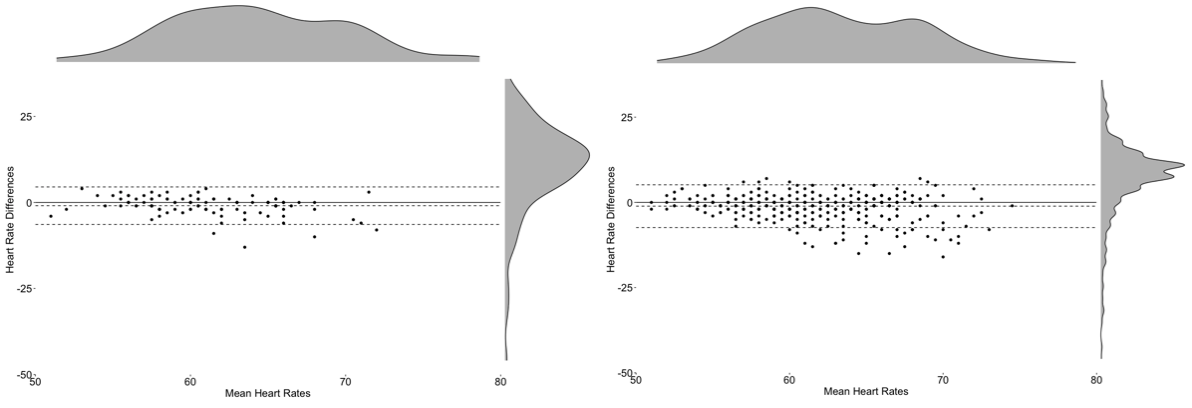
Bland-Altman plots by daily activity. Left: Apple Watch 3 during sleep; right: Fitbit Charge 2 during sleep.

During sitting conditions, the Apple Watch 3 had an ME of −2.47 bpm (lower LoA-upper LoA: −16.94 to 12.01 bpm) and an MAE of 5.24, whereas the Fitbit Charge 2 had an ME of −4.69 bpm (lower LoA-upper LoA; −14.29 to 4.91 bpm) and an MAE of 5.93. During walking conditions, the Apple Watch 3 had an ME of 0.11 bpm (lower LoA-upper LoA: −14.18 to 14.41 bpm) and an MAE of 4.77, whereas the Fitbit Charge 2 had an ME of −6.85 bpm (lower LoA-upper LoA: −28.51 to 14.81 bpm) and an MAE of 9.55. During the running condition, the Apple Watch 3 had an ME of 1.77 bpm (lower LoA-upper LoA: 9.78 to 13.33 bpm) and an MAE of 4.05, whereas the Fitbit Charge 2 had an ME of −14.73 bpm (lower LoA-upper LoA: −29.77 to 0.31 bpm) and an MAE of 14.73. During ADL, the Apple Watch 3 had an ME of −8.50 bpm (lower LoA-upper LoA: −33.78 to 16.78 bpm) and an MAE of 11.74, whereas the Fitbit Charge 2 had an ME of −3.73 bpm (lower LoA-upper LoA: −19.88 to 12.41 bpm) and an MAE of 7.05. Finally, during the sleep condition, the Apple Watch 3 had an ME of −0.95 bpm (lower LoA-upper LoA: −6.39 to 4.50 bpm) and an MAE of 1.96, whereas the Fitbit Charge 2 had an ME of −1.11 bpm (lower LoA-upper LoA: −7.28 to 5.17 bpm) and an MAE of 2.15 (see Table 3 for device error and Bland-Altman statistics and Figures 5-9 for Bland-Altman plots by activity type).

**Table 3:** Device error statistics and Bland-Altman analyses.

Activity and device		Device error		Bland-Altman analysis	
		Mean absolute error	Mean error (SD)	Lower LoA^a^	Upper LoA
**24 hours**					
	Apple Watch 3	4.72	−1.80 (7.40)	−16.31	12.71
	Fitbit Charge 2	4.71	−3.47 (6.17)	−15.55	8.62
**Sitting**					
	Apple Watch 3	5.24	−2.47 (7.39)	−16.94	12.01
	Fitbit Charge 2	5.93	−4.69 (4.90)	−14.29	4.91
**Walking**					
	Apple Watch 3	4.77	0.11 (7.29)	−14.18	14.41
	Fitbit Charge 2	9.55	−6.85 (11.05)	−28.51	14.81
**Running**					
	Apple Watch 3	4.05	1.77 (5.90)	−9.78	13.33
	Fitbit Charge 2	14.73	−14.73 (7.67)	−29.77	0.31
**Activities of daily living**					
	Apple Watch 3	11.74	−8.50 (12.90)	−33.78	16.78
	Fitbit Charge 2	7.05	−3.73 (8.24)	−19.88	12.41
**Sleeping**					
	Apple Watch 3	1.96	−0.95 (2.78)	−6.39	4.50
	Fitbit Charge 2	2.15	−1.11 (3.20)	−7.28	5.17

^a^LoA: limit of agreement.

### Concordance Class Correlation

Overall, across the 24-hour recording, the Apple Watch 3 (CCC=.955, 95% CI 0.945-0.963) and the Fitbit Charge 2 (CCC=.906, 95% CI 0.896-0.914) had strong agreement with the reference method. During sitting conditions, the Apple Watch 3 (CCC=.453, 95% CI 0.321-0.567) had weak agreement and the Fitbit Charge 2 (CCC=.561, 95% CI 0.515-0.603) had moderate agreement with the reference method. During all walking activities, the Apple Watch 3 (CCC=.871, 95% CI 0.807-0.915) and the Fitbit Charge 2 (CCC=.740, 95% CI 0.645-0.812) had strong agreement with the reference method. During the running condition, the Apple Watch 3 (CCC=.864, 95% CI 0.731-0.934) had strong agreement with the reference method, whereas the Fitbit Charge 2 (CCC=.490, 95% CI 0.268-0.663) had weak agreement with the reference method. During the ADL condition, the Apple Watch 3 (CCC=.460, 95% CI 0.204-0.656) had weak agreement with the reference method, whereas the Fitbit Charge 2 (CCC=.739, 95% CI 0.676-0.791) had strong agreement with the reference method. Finally, during the sleep condition, the Apple Watch 3 (CCC=.791, 95% CI 0.715-0.849) and the Fitbit Charge 2 (CCC=.745, 95% CI 0.707-0.779) had strong agreement with the reference method.

## Discussion

### Principal Findings

This study provided the first continuous and ecologically valid assessment of the accuracy of the Apple Watch 3 and the Fitbit Charge 2 as they were devised to be used by consumers (ie, during ecologically valid daily activities) during a 24-hour paradigm of consumer device use conditions.

In line with previous controlled laboratory research [4,5,7-9,11,12], our findings indicated that both wearable devices provided acceptable overall aggregated accuracy (<10% MAPE) across the 24-hour recording period as well as during each type of activity, except for the Apple Watch 3 during ADL. In addition, in line with previous research, both the Apple Watch 3 and the Fitbit Charge 2 slightly underestimated HR across the 24-hour study as compared with ECG and other reference methods [3,4,6,8-11], although this underreporting of absolute HR is unlikely to be problematic in most contexts as this was less than 5 bpm. Although these wearables slightly underestimated HR when values were aggregated by activity, there were a number of individual observations that were inaccurate by significantly large margins, which would be problematic in some contexts (eg, medical settings). This has potential implications for liability of device usage in medical settings [47], indicating that although overall summary statistics may be very accurate for research purposes, any single observation in real time may have a large degree of error, which could be significant for moment-to-moment observations in medical settings. In addition, we found it surprising that the Apple Watch 3 had such a high MAPE (13.70%) during ADL as compared with the Fitbit Charge 2 MAPE (8.29%). This difference was likely because of the fact that the Apple Watch 3 was worn on the dominant hand, which may have made more erratic movements than the Fitbit Charge 2 on the nondominant hand during ADL. In other words, this may have potentially moved the position of the wearable more frequently on the dominant hand, making it more difficult for the PPG sensor to assess and accurately measure HR, as has been found in prior studies [18].

Overall, the Apple Watch 3 had acceptable error across the entire 24-hour period as well as all activities except when the error rate rose above the ±10% threshold for the Apple Watch 3 during ADL (13.70%), while the Fitbit Charge 2 had acceptable error across the entire 24-hour period as well as all activities, although its error got close to the ±10% threshold during walking (9.21%) and running (9.88%). In addition, both devices slightly underestimated heart rate. Finally, as movement became more erratic during certain conditions and as HR increased, the devices became less accurate.

### Strengths and Limitations

This study had a number of strengths that addressed 4 current limitations in wearable studies: this study (1) used a gold standard comparison method for movement within daily life––ambulatory ECG, (2) reported firmware numbers, (3) increased the ecological validity of wearable HR accuracy by taking place during actual consumer device use conditions across a 24-hour period, and (4) took place within an individual, rather than a traditional group of research participants, which creates a novel rapid response design to quickly assess initial wearable HR accuracy in order for the research cycle to keep pace with technological advancements while also controlling for between-subject variability and most potential confounding variables, which allowed for the wearable devices to be the only study variable that varied, thus, providing a powerful test of device accuracy. Furthermore, prior research has shown that there is a 24-hour circadian rhythm to HR [48] and that this can be particularly important as adverse cardiovascular events such as heart attacks, stroke, and cardiac deaths tend to occur in the late mornings [49]. The approach of this study also captured the 24-hour circadian rhythm HR from 3 different devices during real-life conditions, which indicates that these devices can detect changes in HR across the day.

In addition to these strengths, there were also a number of limitations. First, the single-subject design limited various participant demographic factors, such as body mass index, skin tone, and wrist circumference, which have been shown to correlate with HR error rate [7,16]. Future studies should attempt to replicate these results across multiple individuals with diverse body mass index, wrist circumference, skin tone, fitness level, and stress level. Another limitation in this study was that the Fitbit Charge 2 and Apple Watch 3 collected HR measurements at different frequencies. Specifically, the Fitbit Charge 2 recorded an HR measurement each minute, whereas the Apple Watch 3 collected continuous HR measurements during walking and running tasks (the average of these measurements was used for each minute in line with prior research [7]) and every 10 min for all other activities. This discrepancy in device sampling rates combined with proprietary underlying algorithms for the way per minute HR is calculated for each wearable device might help account for the lower reported accuracy of the Apple Watch 3 during the ADL condition. In addition, the single-subject design combined with the Apple Watch 3 sampling rate of approximately every 10 min led to a small number of observations for some conditions. Although continuous recording was not activated on the Apple Watch 3 to approximate real-world usage conditions, future studies should aim to collect larger numbers of subjects to increase the observations for each condition and potentially activate continuous recording on this device. Similarly, although this study had the strength of providing the first 24-hour continuous and ecologically valid assessment of wearable accuracy in real-world conditions, this was also a limitation as this design inherently could not take place within more controlled laboratory settings that used a stationary ECG, rather than an ambulatory ECG that may introduce some additional error. In fact, the running condition had to take place on a treadmill to keep the ECG device stable enough to prevent excessive artifacts. Another limitation of this study is that although that overall error rates of both devices were low, there were some individual observations that were inaccurate by significantly large margins. This indicates that although overall summary statistics for conditions may be very accurate, any single observation in real time may have a large degree of error. Researchers should keep this in mind when using wearable devices in research settings, and this finding emphasizes the importance of data cleaning. Implementing these devices in research settings would likely benefit from automated outlier detection and deletion techniques as would the underlying scoring algorithms. Finally, this study did not counterbalance wrist placement of the wearables to rule out potential influences of wrist circumference, musculature, or movement on the accuracy of HR readings. The subject was right-handed, and therefore, the lower accuracy of the Apple Watch 3 as compared with the Fitbit Charge 2 during the ADL condition may have been because of more erratic wrist motions that accompany many activities in this condition as prior research has indicated that the lack of smooth wrist movements introduces larger HR measurement error [10]. Future studies should provide both between-subjects analyses and within-subjects analyses with devices on both wrists to assess the accuracy of wearables, as hand dominance may influence accuracy.

### Conclusions

This study provided the first continuous and ecologically valid assessment of the accuracy of the Apple Watch 3 and the Fitbit Charge 2 HR measurements as they were devised to be used by consumers out in the real world during a 24-hour paradigm of actual consumer device use conditions. Overall, both the Apple Watch 3 and Fitbit Charge 2 had acceptable HR accuracy when aggregated overall across the 24-hour period and during each condition, except for the Apple Watch 3 during the ADL condition. In addition, both the Apple Watch 3 and Fitbit Charge 2 slightly underestimated HR. Furthermore, both erratic wrist movements and higher HR were associated with lower device accuracy. It is important to note that although overall HR accuracy statistics for most conditions were acceptable, there were a number of individual observations that varied widely from the gold standard ECG, which indicates that any single measurement viewed in real time cannot be interpreted as an accurate measurement that has implications for medical liability of device usage [47]. Overall, wearable devices likely will not be replacing the gold standard ECG in a medical setting anytime soon, but both the Apple Watch 3 and the Fitbit Charge 2 can be used to supplement these gold standard methods in research and clinical applications. They may be particularly useful in big data studies as these devices had acceptable error rate in almost all activities while being relatively cheap, mobile, unobtrusive, and scalable as compared with gold standard medical equipment.

## Abbreviations

ADL: activities of daily living
bpm: beats per minute
CCC: concordance class correlation
ECG: electrocardiogram
HR: heart rate
LoA: limit of agreement
MAE: mean absolute error
MAPE: mean absolute percent error
ME: mean error
OSF: Open Science Framework
PPG: photoplethysmography
